# Magnesium ascorbyl phosphate vesicular carriers for topical delivery; preparation, in-vitro and ex-vivo evaluation, factorial optimization and clinical assessment in melasma patients

**DOI:** 10.1080/10717544.2022.2036872

**Published:** 2022-02-14

**Authors:** Soha M. Kandil, Iman I. Soliman, Heba M. Diab, Nermeen I. Bedair, Marwa H. Mahrous, Ebtsam M. Abdou

**Affiliations:** a Department of Pharmaceutics and Industrial Pharmacy, Faculty of Pharmacy, Modern University of Technology and Information (MTI), Cairo, Egypt; b Department of Pharmaceutics and Industrial Pharmacy, Faculty of Pharmacy, Cairo University, Cairo, Egypt; c Department of Dermatology, Venereology and Andrology, Faculty of Medicine, Ain Shamas University, Cairo, Egypt; d Department of Dermatology, Andrology, Sexual Diseases and STDs, Faculty of Medicine, Helwan University, Helwan, Egypt; e Department of Pharmaceutics, National Organization of Drug Control and Research (NODCAR), Giza, Egypt

**Keywords:** Antera 3D^^®^^ camera, ethosomes, hyperpigmentation, magnesium ascorbyl phosphate, melasma, niosomes, permeation, skin

## Abstract

Ascorbic acid (vitamin C) is an antioxidant that is widely used in cosmetics in skincare products. Due to the excessive low stability of ascorbic acid in cosmetic formulations, the stabilized ascorbic acid derivative, magnesium ascorbyl phosphate (MAP) was formulated as vesicular carriers; ethosomes and niosomes. The aim was to deliver MAP at the intended site of action, the skin, for sufficient time with enhanced permeation to get an effective response. Ethosomes were formulated using a full 3^2^ factorial design to study ethanol and phospholipid concentration effect on ethosomes properties. Niosomes were formulated using 2^3^ factorial designs to study the effect of surfactant type, surfactant concentration and cholesterol concentration on niosomes properties. The prepared formulations were evaluated for their Entrapment efficiency, particle size, polydispersity index, zeta potential and % drug permeated. The optimized ethosomal and niosomal formulations were incorporated into carbopol gel and evaluated for their permeation, skin retention and stability. A comparative split-face clinical study was done between the ethosomal and niosomal formulations for melasma treatment using Antera 3 D^®^ camera. The optimized ethosomal and niosomal gels showed comparable controlled permeation and higher skin retention over their ethosomes and niosomes formulations respectively. Magnesium ascorbyl phosphate ethosomal gel showed clinically and statistically significant melanin level decrease after one month while MAP niosomal gel showed clinically and statistically significant melanin level decrease after six months. A combination of MAP ethosomes and niosomes could be promising skincare formulations for melasma and hyperpigmentation short and long-term treatment.

## 1. Introduction

The supportive care of skin hyperpigmentation and melasma treatment has dramatically improved over the past two decades. Melasma is an acquired hyperpigmentation disease that commonly affects middle-aged patients particularly those with dark complexion . It occurs with stains in shades of brown to bluish-gray, with irregular borders and located in more photo-exposed areas, it usually affects the face and neck (Cestari et al., 2014).

It is known that vitamin C (ascorbic acid), one of the naturally occurring antioxidants in nature, is a vital antioxidant agent because of its free radical scavenging ability and its importance for the synthesis of collagen (Telang, 2013).

The absorption of vitamin C in the gut is limited by an active transport mechanism and hence a little amount is absorbed although a high oral dosage is taken (Lykkesfeldt & Tveden-Nyborg, 2019). In addition, vitamin C bioavailability in the skin is inadequate when it is administered orally (Talakoub et al., 2009). So, it is more favorable to use vitamin C as topical formulations in the practice of dermatology.

Unfortunately, vitamin C has problems of poor stability, easy degradation in oxidation pathways and poor penetration into the skin. Also, a high concentration of ascorbic acid will cause irritation especially for sensitive skin (Apridamayanti et al., 2016). Instead, they use of stable esterified derivatives of ascorbic acid, vitamin C, is being explored (Al-niaimi & Chiang, 2017).

Magnesium ascorbyl phosphate (MAP) is the most stable, water-soluble preferred ascorbic acid derivative, ascorbyl ester. This molecule is easily absorbed into the skin and has a hydrating effect on the skin and decreases transepidermal water loss. It is also a free radical scavenger that is photoprotective and increases collagen production under laboratory test conditions (Telang, 2013). MAP was formulated into different topical formulations such as aspasomal cream (Aboul-Einien et al., 2020), topical skin-care cream (o/w emulsion) (Smaoui et al., 2013) and nanoemulsion spray and cream (Karsono & Harahap, 2016).

Among the different micro and nano-systems formulated for drug encapsulation and transdermal delivery, ethosomes represent a smart strategy. Ethosomes were first developed for transdermal delivery by Elka Touitou as soft malleable lipo-vesicle carriers containing phospholipid with high ethanol and water concentration (Touitou, 2002). Ethosomes are characterized by their simple preparation with prolonged efficacy and safety as they show no significant irritation to human skin and cause no toxicity to the cells in vitro (Godin & Touitou, 2005; Paolino et al., 2005). In addition, they have the ability to efficiently entrap various hydrophilic, lipophilic and amphiphilic molecules due to their high degree of lamellarity and the presence of ethanol in the vesicles that allows for better solubility of many drugs (Imam et al., 2017; Tiwari et al., 2018).

Niosomes are vesiclular systems made from self-organizing nonionic surfactant systems with or without the addition of cholesterol and other lipid constituents with subsequent hydration in aqueous media (Kumar et al., 2013). They can encapsulate both hydrophilic and hydrophobic drugs either in an aqueous layer or in a vesicular membrane made of lipid material (Khan et al., 2019), they are extensively used for transdermal drug delivery.

The main aim of the study was to use Magnesium ascorbyl phosphate (MAP), instead of ascorbic acid, to be formulated into two different vesicular drug delivery systems, ethosomes and niosomes, to deliver it in sufficient concentration at the site of action, the skin, for a long time with enhanced permeation for effective response and treatment.

## 2. Materials and methods

### 2.1. Materials

Magnesium ascorbyl phosphate (MAP) was purchased from SOST (Xi’an Sost Biological Science and Technology Co., Ltd. Xi’an, China). Phosphatidylcholine (PC) from soybean lecithin, containing not less than 94% PC) from Alfa Aesar. Ethyl alcohol absolute 99% and propylene glycol from United Company for Chemistry and Medical Preparation, Cairo, Egypt. Carbopol 934 was obtained from ADWIC (El Nasr Pharmaceutical Chemicals Co., Cairo, Egypt). Sorbitan monostearate (Span 60) Sorbitan monooleate (Span 80) and Cholesterol (Chol) were purchased from Loba Chemie, Mumbai, India. All other ingredients were of analytical grade.

### 2.2. Preparation of magnesium ascorbyl phosphate – loaded ethosomes

Magnesium ascorbyl phosphate-loaded ethosomes were prepared using the cold method as described previously (Hongdan et al., 2018; Dina et al., 2020). Phosphatidylcholine and MAP were dissolved in an ethanol-propylene glycol mixture and stirred using a magnetic stirrer (Wisestir, Wisd Lab. Instruments, USA) until complete dissolution. The mixture was heated to 30 °C in a water bath. The double-distilled water, pre-heated to 30 °C, was slowly injected using a syringe pump with constant stirring at 700 rpm in a closed vessel for 5 min. The system was kept at 30 °C throughout the preparation. The resulted dispersion was sonicated at 4 °C using a probe sonicator (Sonifier^®^ 250 Branson, USA) for three cycles; each is of 5 min with 5 min rest between the cycles. Formulations were kept at 4 °C for further evaluation.

### 2.3. Preparation of magnesium ascorbyl phosphate – loaded niosomes

Magnesium ascorbyl phosphate-loaded niosomes were prepared using the previously described thin-film hydration method (Yeo et al., 2019). The determined amount of surfactant and cholesterol were dissolved in 10 ml chloroform in a round-bottomed flask. The solvent was evaporated under reduced pressure using rotary evaporator (Rotavap, Type R 110, Buchi, Switzerland) at 150 rpm and a temperature of 60 ± 2 °C until thin film was formed.

The film was hydrated with 10 mL buffer solution (pH 7.4) containing the determined MAP amount and pre-heated to 50 ± 2 °C with the aid of small glass beads (8 small glass beads, each of a diameter of 4 mm) to facilitate the film hydration until the suspension is formed. The suspension was cooled at room temperature and then subjected to sonication using a probe sonicator (Sonifier^®^ 250 Branson, USA) in an ice-bath for three intermitted intervals each one for 5 minutes. The prepared niosomal dispersion was kept in the refrigerator at 4 °C for further evaluation.

### 2.4. Statistical design of the magnesium ascorbyl phosphate -loaded ethosomes

A 3^2^ full factorial design was employed to statistically investigate the effect of different formulation variables on the properties of the prepared MAP-loaded ethosomes using Design-Expert^®^ software (version 7; Stat-Ease, Inc., Minneapolis, MN). Two independent factors were screened at three different levels as follows: Ethanol concentration (X1) at 30, 40, and 50% w/v and PC concentration (X2) at 1, 2, and 3% w/v. Five selected independent variables were evaluated as follow: Entrapment efficiency (EE%) (Y1), Particle size (PS) (Y2), Polydispersity index (PDI) (Y3), Zeta potential (ZP) (Y4), and MAP % permeated after 8 hours (Y5). The design parameters and experimental runs coded as FE1: FE9, are shown in Table 1. MAP (0.3% w/v) and PG (10% v/v) were kept constant in all formulations. A sufficient quantity of water was used to complete the volume up to 100% v/v.

**Table 1.** Experimental runs, independent and dependent variables of the 3^2^ full factorials experimental design of Magnesium ascorbyl phosphate-loaded ethosomes.

Runs*	Factors (Independent variables)				Responses (Dependent variables)				
	Ethanol % (v/v) (mL/100 mL)		PC % (w/v) (g/100 mL)		Y1:E.E (%)	Y2: Particle size (nm)	Y3:PDI	Y4:ZP (mV)	Y5: % permeated (8 h)
FE1	(−1)	30	(−1)	1	51.45 ± 2.34	148.62 ± 15.27	0.496 ± 0.071	−32.24 ± 5.24	38.12 ± 2.61
FE2	(−1)	30	(0)	2	65.56 ± 1.56	158.73 ± 18.53	0.482 ± 0.054	−33.42 ± 3.45	33.24 ± 1.93
FE3	(−1)	30	(1)	3	60.71 ± 2.04	167.42 ± 12.37	0.480 ± 0.123	−31.47 ± 5.34	31.80 ± 2.52
FE4	(0)	40	(−1)	1	63.43 ± 3.17	157.27 ± 17.63	0.415 ± 0.142	−29.37 ± 3.74	51.62 ± 2.14
FE5	(0)	40	(0)	2	70.32 ± 2.86	163.47 ± 10.46	0.375 ± 0.085	−26.72 ± 2.64	42.83 ± 3.51
FE6	(0)	40	(1)	3	66.67 ± 3.46	183.37 ± 20.46	0.262 ± 0.053	−30.28 ± 4.72	40.92 ± 2.13
FE7	(1)	50	(−1)	1	83.43 ± 2.23	160.57 ± 13.74	0.257 ± 0.008	−35.91 ± 4.08	68.47 ± 3.60
FE8	(1)	50	(0)	2	86.14 ± 1.58	180.63 ± 11.42	0.237 ± 0.014	−32.92 ± 3.81	61.91 ± 2.94
FE9	(1)	50	(1)	3	76.47 ± 3.51	193.52 ± 10.58	0.222 ± 0.047	−36.64 ± 3.64	57.52 ± 2.50

*MAP (0.3% w/v (0.333 g/100 mL)) and PG (10% v/v (10 mL/100 mL)) were kept constant in all formulations, volume was completed up to 100 mL with distilled water. EE: Entrapment Efficiency, PDI: Polydispersity index, ZP: Zeta potential.

### 2.5. Statistical design of the magnesium ascorbyl phosphate -loaded niosomes

Magnesium ascorbyl phosphate niosomes were prepared using 2^3^ full factorial design, Design-Expert^®^ software (version 7; Stat-Ease, Inc., Minneapolis, MN), in order to study the effect of different formulation variables on the physicochemical properties of the prepared vesicles. The three independent factors were: Surfactant type (X1), surfactant molar ratio (X2), and Cholesterol (Chol) molar ratio (X3). The selected independent variables were: Entrapment efficiency (EE%) (Y1), Particle size (PS) (Y2), Polydispersity index (PDI) (Y3), Zeta potential (ZP) (Y4), and MAP % permeated after 8 hours (Y5). The design parameters and experimental runs coded as FN1: FN8, are shown in Table 2. MAP one molar ratio was kept constant in all formulations.

**Table 2.** Experimental runs, independent and dependent variables of the 2^3^ full factorials experimental design of Magnesium ascorbyl phosphate-loaded niosomes.

Runs*	Factors (Independent variables)					Responses (Dependent variables)				
	Surfactant type	Surfactant molar ratio		Cholesterol molar ratio		Y1:EE (%)	Y2: Particle size (nm)	Y3:PDI	Y4:ZP (mV)	Y5: % permeated (8 h)
FN1	Span 60	(−1)	1	(−1)	1	69.80 ± 3.56	218.60 ± 22.52	0.253 ± 0.124	−26.83 ± 3.34	36.67 ± 3.64
FN2	Span 60	(−1)	1	(+1)	2	78.63 ± 2.47	258.23 ± 16.85	0.281 ± 0.092	−32.42 ± 2.58	30.14 ± 1.96
FN3	Span 60	(+1)	2	(−1)	1	86.82 ± 4.52	138.43 ± 17.84	0.312 ± 0.112	−27.56 ± 2.67	54.42 ± 2.35
FN4	Span 60	(+1)	2	(+1)	2	92.13 ± 3.09	169.32 ± 12.22	0.292 ± 0.163	−31.42 ± 2.43	46.37 ± 3.24
FN5	Span 80	(−1)	1	(−1)	1	53.85 ± 4.52	362.43 ± 24.63	0.415 ± 0.131	−25.82 ± 4.52	33.34 ± 2.05
FN6	Span 80	(−1)	1	(+1)	2	61.24 ± 2.68	384.62 ± 14.52	0.281 ± 0.082	−32.63 ± 3.84	28.46 ± 2.98
FN7	Span 80	(+1)	2	(−1)	1	68.32 ± 2.97	278.40 ± 10.58	0.354 ± 0.028	26.08 ± 3.06	50.58 ± 2.68
FN8	Span 80	(+1)	2	(+1)	2	87.32 ± 3.12	315.62 ± 11.23	0.382 ± 0.036	−30.52 ± 2.41	40.42 ± 3.21

*MAP (one molar ratio) was kept constant in all formulations. EE: Entrapment Efficiency, PDI: Polydispersity index, ZP: Zeta potential.

### 2.6. Evaluation of the prepared magnesium ascorbyl phosphate -loaded ethosomes and niosomes

#### 2.6.1. Entrapment efficiency (EE%) measurements

The un-entrapped MAP was separated from the prepared ethosomal and niosomal suspensions through ultracentrifugation using a cooling centrifuge (Model 8880, Centurion Scientific Ltd., W. Sussex, UK) at 4 °C and 15,000 rpm for 2 hours. Further ultracentrifugation was done to the supernatant to separate any suspended vesicles while the residue was washed three times each time with one-milliliter phosphate-buffered saline (pH 7.4) (Sujatha et al., 2016).

The supernatant was collected, properly diluted, and measured spectrophotometrically at a wavelength of 251 nm using Shimadzu spectrophotometer (Model UV- 1601, Japan). The residue was disrupted using 5 mL ethanol, filtered, diluted with phosphate buffer and measured spectrophotometrically at a wavelength of 251 nm to determine the MAP content using a previously constructed MAP calibration curve.

Entrapment efficiency was determined by both direct and indirect methods using the following equations:

(1) For direct method: EE% = MAP amount in the vesicles/MAP amount used in formulation × 100

(2) $$\begin{array}{c} \text{For}\;\text{indirect}\;\text{method}:\;\text{EE}\%=\;\left(\text{MAP}\;\text{amount}\;\text{used}\;\text{in}\;\text{formulation}-\text{MAP}\;\text{free}\;\text{amount}\right)/\text{MAP}\\ \text{amount}\;\text{used}\;\text{in}\;\text{formulation} \end{array}$$

Each experiment was done in triplicates and results were expressed as mean values ± SD.

#### 2.6.2. Particle size, polydispersity index (PDI) and zeta potential measurement

Particle size analysis (mean particle size and polydispersity index (PDI)) and zeta potential measurement of the prepared MAP-loaded ethosomes and niosomes were performed using dynamic light scattering (Zeta-sizer Nano ZS-90, Malvern Instruments, Worcestershire, UK). The samples were appropriately diluted with distilled deionized water to avoid a multi-scattering phenomenon. All measurements were carried out in triplicates and results were expressed as mean values ± SD.

#### 2.6.3. *Ex vivo* permeation of MAP from the prepared ethosomes and niosomes

This study was conducted in accordance with the National Institutes of Health guide for the care and use of laboratory animals and its protocol was approved by The Cairo University Protection of Experimental Animals Committee (Approval code: P.T4.1.2).

For this permeation study, the abdominal skin of newly born male Wistar albino rats weighing 100–150 g was used. The animals were first mercy sacrificed through prolonged inhalation of chloroform. After sacrificing, the abdominal skin was excised after careful shaving of the abdominal area. The subcutaneous tissue was surgically removed then the dermis side was wiped with a cotton swab three times and wetted with isopropyl alcohol. The full skin specimen was washed with distilled water, cut into pieces of appropriate size, wrapped in aluminum foil and stored in a freezer till use (Aboul-Einien et al., 2020).

Before the experiment, the skin was thawed at room temperature and examined visually under intense light for any damage or holes and any damaged skin was discarded. The prepared skin specimens were mounted onto Franz diffusion cell (Erweka HDT6, Germany), after being soaked in phosphate-buffered saline (pH 7.4) for 30 minutes at room temperature, between the donor and receptor compartments with the dermal side toward the receptor compartment.

A volume of 20 mL of phosphate-buffered saline (pH 7.4) was placed in the receptor compartment and the whole assembly was mounted in a water bath which was thermostatically adjusted at a temperature of 32 ± 0.5 °C and stirred at 50 rpm.

Weighed amount (200 mg) of MAP-loaded ethosomes or niosomes was applied to the skin surface which had an available diffusion area of 2.5 cm^2^. Sink conditions were maintained in the receptor compartment. Three- milliliters of samples of the receptor medium were removed at appropriate time intervals (0.05. 1. 2. 3. 4. 5. 6, 8 and 24 h) and immediately replaced with fresh medium pre-heated to 32 ± 0.5 °C. Samples were measured spectrophotometrically at a wavelength of 251 nm (Shimadzu spectrophotometer, Model UV- 1601, Japan) for the determination of MAP concentration. Each experiment was done in triplicates and results were expressed as mean ± SD.

### 2.7. Preparation of MAP-loaded ethosomal and niosomal gel

Carbopol gel (1% w/v) was prepared as described earlier (Aggarwal & Goindi, 2013). 500 mg Carbopol 934 was dispersed in 50 mL distilled water for 2 h and then stirred using a magnetic stirrer (Wisestir, Wisd Lab. Instruments, USA) at 600 rpm. The pH of the dispersion was adjusted to 7 ± 0.2 using (0.3–0.5 mL) of triethanolamine with continuous stirring until a transparent gel is formed. The gel was left overnight for complete gelation and de-aeration. The calculated amount of MAP-loaded ethosomes (FE7) or niosomes (FN3) was added to Carbopol gel, to give the final concentration of MAP of 5% w/w, through gentle mixing until homogenous ethosomal and niosomal gels were obtained.

### 2.8. Transmission electron microscopy (TEM)

Transmission electron microscope (TEM) (JEOL JEM1230, Tokyo, Japan) was used to undergo the morphological examination of the optimized ethosomal and niosomal formulation. A drop of the sample was placed on a carbon-coated copper grid to leave a thin film that was negatively stained with 1% phosphotungstic acid (PTA). The grid was left to dry, and samples were scanned under the transmission electron microscope operating at an accelerating voltage of 80 kV.

### 2.9. *Ex-vivo* skin permeation and skin retention studies of MAP from the optimized formulations and the prepared ethosomal and niosomal gels

Skin permeation studies of MAP through rat skin from the optimized formulations and their prepared gels in addition to MAP solution (equivalent to 10 mg MAP in buffer solution, pH 7.4) were done using the same method as described above in section Ex vivo permeation of MAP from the prepared ethosomes and niosomes.

Retention studies were done to determine the amount of drug deposited into the skin layers after time intervals of 8 and 24 hours. For each tested formulation in this study, plain one having the same components except the drug was prepared to be used as a control and tested in the same method. At the predetermined time intervals, the skin membrane was removed, rinsed three times with distilled water and gently blotted with a paper towel between rinses. The excess skin was removed and the effective diffusion area was separated, cut into small pieces and weighed. The skin specimen was then soaked in 10 mL distilled water/methanol 1:1 mixture and homogenized using tissue homogenizer for 2 cycles, each of 10 min, at 10,000 rpm (Thomas VR Scientific Tissue homogenizer, USA) (Kelchen & Brogden, 2018; Aboul-Einien et al., 2020).

The homogenate was then centrifuged at 4 °C using a cooling centrifuge (Model 8880, Centurion Scientific Ltd., W. Sussex, UK), filtered, and the supernatant was measured spectrophotometrically to determine MAP concentration. Each formulation was measured against its control as a blank. The experiment was repeated three times and the mean values, as well as standard deviations, were calculated. Results from permeation and retention studies were subjected to statistical analysis using one-way analysis of variance test at a level of significance of *p* < .05

### 2.10. Stability studies

Samples of the optimized formulations (FE7 & FN3) were stored in the tightly closed vial at two different conditions; the first was at 4 °C with controlled humidity of 75% RH and the second was at room temperature. Samples were taken at time intervals of 0, 1, 2 and 3 months, stability was assessed through EE% and particle size measurements in addition to visual examination for any color change or sedimentation.

### 2.11. Clinical assessment of MAP ethosomal and niosomal gels efficacy in treating melasma using ANTERA 3 D^®^ camera through split-face comparative study

#### 2.11.1. Patients and methods

Recruitment of participants:

This comparative split-face study included 40 patients with bilateral acquired facial hyperpigmentation and melasma recruited from the Dermatology outpatient clinic, Demerdash hospitals, between February 2020 and January 2021. The study was conducted according to the Declaration of Helsinki Principles and was approved by the Research Ethical Committee, Faculty of Medicine, Ain Shams University, and fulfilled all the ethical aspects required in human research. All patients received full information about the description of the treatment, possible side effects and they all provided informed consent. We excluded patients who received any cosmetic treatment within the last 6 months prior to enrollment, pregnant and lactating women, women on any hormonal contraception, patients with any photo-aggravating conditions such as systemic lupus erythematosus as well as patients with any skin disorder other than hyperpigmentation.

Full history was obtained from all candidates before a thorough dermatological examination. The skin was examined and photographed using Antera 3 D^®^ Camera (Miravex, Dublin, Ireland). Evaluation of treatment response was done objectively using the Antera 3 D^®^ camera, at baseline (0) then at 1 and 6 months. In the current study; the average melanin concentration mode of the camera was used for the assessment of skin pigmentation.

Every patient was given 2 different containers labeled as right (Rt) and left (Lt). The right (Rt) labeled container contains MAP niosomal gel and the left (Lt) one contains MAP ethosomal gel each with 5% w/w MAP concentration as reported previously (Murtaza et al., 2016). Patients were instructed to apply a fingertip amount from each container to the hyperpigmented lesion on each side as mentioned on the label, once at night daily for 6 months. Patients were instructed to apply a broad-spectrum sunscreen that protects against both UVA and UVB and to report any side effects in the form of erythema, itching or burning.

#### 2.11.2. Statistical analysis

The computer program IBM SPSS (Statistical Package for the Social Science; IBM Corp, Armonk, NY, USA) release 22 for Microsoft Windows was done for all statistical calculations. All the data were described as the mean ± standard deviation (± SD), median and range, or frequencies (number of cases) and percentages when appropriate. Comparison between Rt and Lt sides as well as overtime was done using paired *t*-test after applying Bonferroni correction for multiple comparisons. Two-sided *p*-values less than .05 were considered statistically significant.

## 3. Results and discussion

### 3.1. Effect of formulations variables on different properties of the prepared magnesium ascorbyl phosphate – loaded ethosomes

Magnesium ascorbyl phosphate–loaded ethosomes were successfully prepared using the classical cold method which is the simplest and most widely used method for the preparation of ethosomal systems as it is a simple production method avoiding high energy input and mechanical stress thus preventing active constituent degradation (Abdulbaqi et al., 2016; Hallan et al., 2020). The prepared MAP-loaded ethosomes were evaluated for several parameters to investigate the effect of different formulation factors on these parameters. All the screened responses were simultaneously fitted to linear, interaction, and quadratic models. All of them followed the quadratic model.

**Figure 1.**
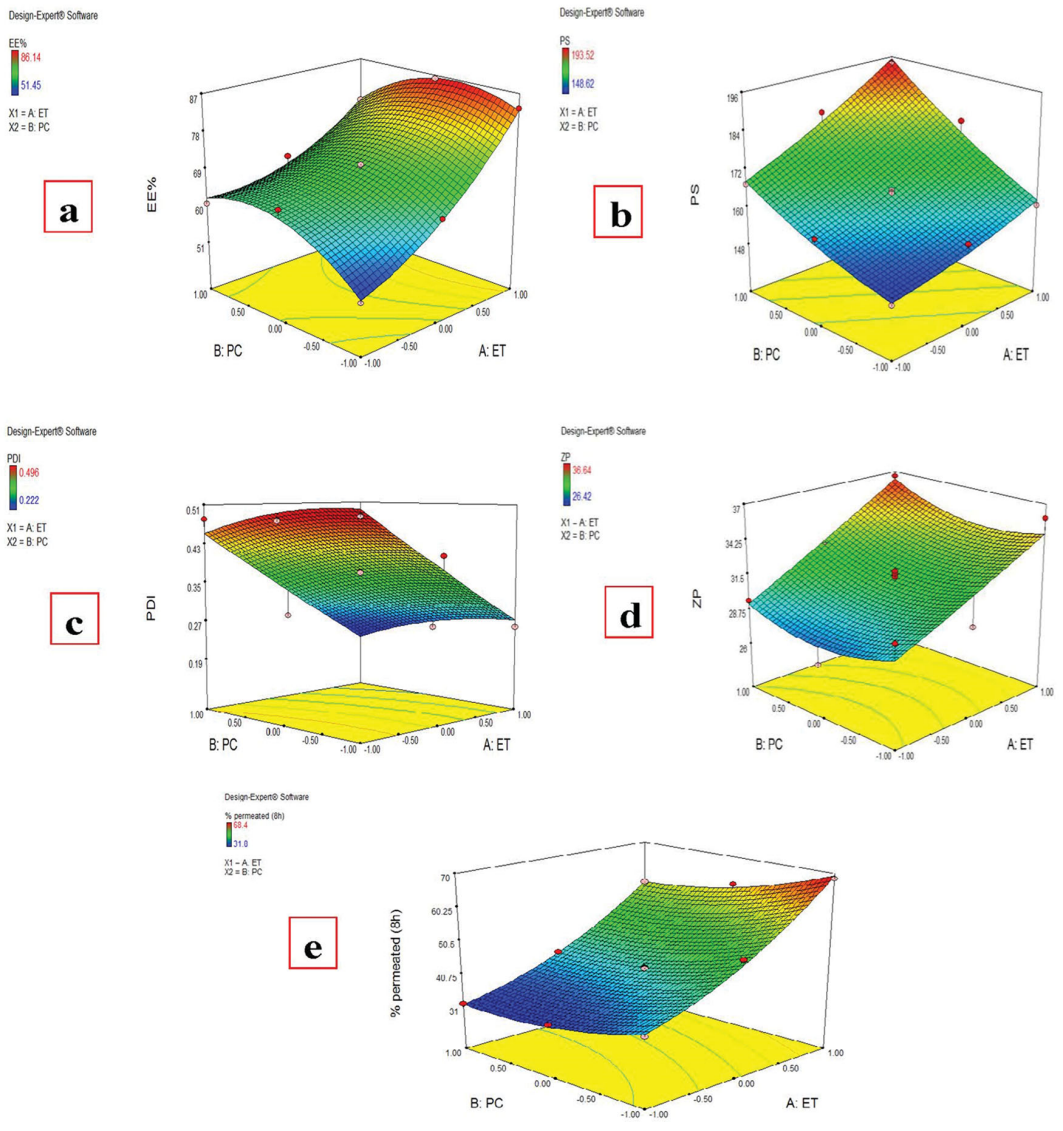
Effect of different independent factors; (X1): Ethanol concentration, (X2): PC concentration on: (a) EE%, (b) PS, (c) PDI, (d) ZP, (e) MAP % permeated (8 h) of the prepared MAP Ethosomes.

Results showed that both Ethanol (X1) and PC (X2) concentrations have a significant effect (*P* < .0001) on MAP EE%, Figure 1(a), with the final equation in terms of coded factors was:

(3) $$\text{EE}\%\;=\;+70.49\;+11.39\;*\text{ A }+0.92*\text{ B }-4.05*\text{ A }*\text{ B }+4.40\;*{\;A}^{2}-6.40\;*{\;B}^{2}$$

Magnesium ascorbyl phosphate entrapment efficiency in the prepared ethosomes was measured by both direct and indirect methods and both methods gave similar results which ranging from 51.45 ± 2.34% to 86.14 ± 1.58%, Table 1. Increasing ethanol ratio from 30% to 40% and finally to 50% resulted in an increase in the MAP EE% at all PC ratios which is in agreement with previous results (Touitou et al., 2000). It is known that ethanol can increase the ethosomal membrane fluidity during ethosomes formation and improve their stability allowing higher encapsulation of the drug (Jain et al., 2007). As MAP is a hydrophilic drug, with higher amounts of ethanol, it could be accumulated in the aqueous core of the vesicles (Barupal et al., 2010; Wang et al., 2013). This is in agreement with a previous report that the entrapment efficiency of the ethosomes containing a water-soluble drug was higher than that of conventional vesicle preparations (Liu et al., 2011).

Increasing the PC ratio from 1 to 2% w/v resulted in a significant increase in MAP EE% at constant ethanol concentrations due to increased vesicle packing and rigidity. Also, at higher PC amounts, more volume of the aqueous phase in which the drug is dissolved is entrapped around lipid bilayer to hydrate PC. On the other side, a further increase of the PC ratio to 3% w/v resulted in a significant decrease in the EE%. This is in accordance with previous reports that beyond a certain concentration, a further increase in phospholipid concentration will not affect the EE% (Zhaowu et al., 2009) or will result in a decrease of the EE% (Dina et al., 2020). This may be attributed to that increase in the PC ratio with a constant amount of the drug is accompanied by a decrease in the drug/lipid ratio with high lipid amount which may squeeze out some of the encapsulated drug resulting in lower EE% (Dina et al., 2020).

Preparing vesicular carriers with small PS is one of the major concerns to enhance MAP permeation through the skin. Vesicular size of the prepared ethosomes ranged from 148.62 ± 15.27 to 193.52 ± 10.58 nm, Table 1, which is relatively suitable for skin penetration as it was reported previously that vesicles up to 300 nm are able to release the medicament in deep layers of skin (Verma et al., 2003).

Both ethanol and PC concentrations were found to have a significant effect on the particle size of MAP-loaded ethosomes, Figure 1(b), with the final equation in terms of coded factors was:

(4) $$\text{PS}=\;+165.43\;+9.99\;*\text{ A }+12.98*\text{ B }+3.54\;*A*\text{ B }+1.43*A^{2}+2.07*{\;B}^{2}$$

Higher ethanol concentrations at constant PC amounts resulted in larger vesicular size in disagreement with many previous studies (Dave et al., 2010; Madhavi et al., 2019). These results may be related to that MAP is a hydrophilic drug that tends to be accumulated into the aqueous phase, unlike lipophilic drug which tends to be encapsulated or adsorbed into the lipid bilayer (Madhavi et al., 2019), so higher ethanol concentration gives higher EE% and consequently larger particle size. Increasing the PC amounts at constant ethanol concentrations resulted in an increase of the particle size due to the increase of phospholipid molecules in the vesicle bilayers.

The PDI of the prepared ethosomes ranged from 0.222 ± 0.047 to 0.496 ± 0.071, Table 1. Significant effect of ethanol concentration was found on the prepared formulations PDI while the non-significant effect of PC concentration was found, Figure 1(c), with the final equation in terms of coded factors was:

(5) $$\text{PDI}\;=\;+0.37\;-0.12\;*\;A-0.034\;\;*\text{ B }-4.750E-003\;*\text{ A }*\text{ B }+3.759E-003*{\;A}^{2}-0.017*{\;B}^{2}$$

Higher PDI values were found with lower ethanol concentrations indicating heterogeneous vesicle size distribution. It was reported that PDI values equal to or smaller than 0.3 are deemed acceptable and indicate a homogenous vesicle size distribution (Chen et al., 2011) which was achieved for all formulations prepared with 50% v/v ethanol.

To determine the long-term physical stability of the colloidal systems, the zeta potential should be evaluated as a higher zeta potential value indicates better stability due to increased electrostatic repulsion which could prevent the vesicles from aggregation (Sujatha et al., 2016). Most formulations had ZP values around or greater than − 30 mv, Table 1, indicating good stability of the prepared ethosomes (Dubey et al., 2007). Only ethanol concentration was found to have a significant effect on the ZP of the prepared ethosomes while PC amounts had no significant effect (*P* value =.4481), Figure 1(d), with the final equation in terms of coded values was:

(6) $$\text{ZP}=31.00+3.22\;*\;A+0.48\;*\;B+0.38*\text{ A }*\;B+0.097\;*{\;A}^{2}+1.25\;*{\;B}^{2}$$

Higher ethanol concentration resulted in a higher ZP value. It was reported that ethanol at high concentration could impart a net negative charge to the ethosomal system and confer it some degree of steric stabilization due to electrostatic repulsion and delaying the formation of aggregates (Tunyaluk et al., 2017).

A significant effect was found for both ethanol and PC amount on the cumulative MAP percent permeated from different MAP-loaded ethosomes after 8 h, Figure 1(e), which ranged from 31.80 ± 2.51 to 68.47 ± 3.60%, Table 1, with the final equation in terms of coded factors was:

(7) $$\%\;\text{permeated}\;\left(8h\right)\;=\;+42.88\;+14.12*\text{ A }-4.65*\;B-1.15*\text{ A }*\text{ B }+3.88*{\;A}^{2}+2.58*{\;B}^{2}$$

Increasing ethanol amount with constant PC ratio led to an increase in the MAP amount permeated through rat skin which agrees with previous results (Tiwari et al., 2018). Ethanol can reduce the interfacial tension of the vesicle membrane providing elasticity of the vesicle membrane and increasing its ability to cross the skin. Also, this effect is related to the solvent effect of Ethanol on skin lipids followed by skin permeation of ethosomes as ethanol partially fluidizes the intercellular lipids and consequently enhances the permeation flux and cumulative drug permeated (Jain et al., 2015). In addition, it was reported that ethosomal vesicles with high ethanol concentrations have thinner membranes (Zhou et al., 2010) allowing the faster release of the drug in the skin layers after permeation.

Increasing PC amount with constant ethanol concentration led to decreasing the MAP amount permeated through rat skin. This may be explained as mentioned by Jain et al. were increasing the PC amount led to more PC packing which increases the thickness of the hydrophobic portion of the bilayer, increases the vesicular rigidity, and decreases the rate of motion of the lipid tails resulting in decreasing MAP release from the vesicles as MAP is already dissolved in the hydrophilic portion of the vesicle (Jain et al., 2015).

### 3.2. Optimization of the prepared magnesium ascorbyl phosphate -loaded ethosomes

After analysis of all responses, constraints (goals) on dependent (responses) and independent variables (factors) using Design-Expert^®^ software (version 7; Stat-Ease, Inc., Minneapolis, MN) was applied for obtaining the optimized formulation. Optimization was done by both methods, numerical and graphical. Optimized formulation was selected based on the criteria of maximum EE%, particle size (in range), PDI < 0.3, ZP > 30 mV, and maximum % permeated. Two solutions were obtained with the first one being of the desirability of 0.956 which matched with FE7 ethosomal formulation composition. So, FE7 was selected as optimized formulation for further evaluation.

### 3.3. Effect of formulation variables on different properties of the prepared magnesium ascorbyl phosphate -loaded niosomes

Magnesium ascorbyl phosphate -loaded niosomes were prepared using the thin-film hydration method which is the most common and extensively used method for niosomes preparation (Ravalika & Sailaja, 2017). During preparation, the temperature was maintained at 60 °C ± 2 which is above the gel-liquid crystal transition temperature (Tc) of the used surfactants to allow hydration of lipids in their fluid phase which has a significant impact on the shape and size of the vesicles and surfactants assembly into them (Kazi et al., 2010; Dua et al., 2012).

Span 60 and Span 80 with low hydrophilic-lipophilic balance (HLB) (4.7 and 4.3 respectively) were selected as nonionic surfactants for niosomes formation as nonionic surfactants presenting a high HLB value hinder the formation of bilayer structure while Span 60 and Span 80 promote the formation of stable, rigid, and intact niosomes with the capability of high entrapment efficiency (Basiri et al., 2017).

**Figure 2.**
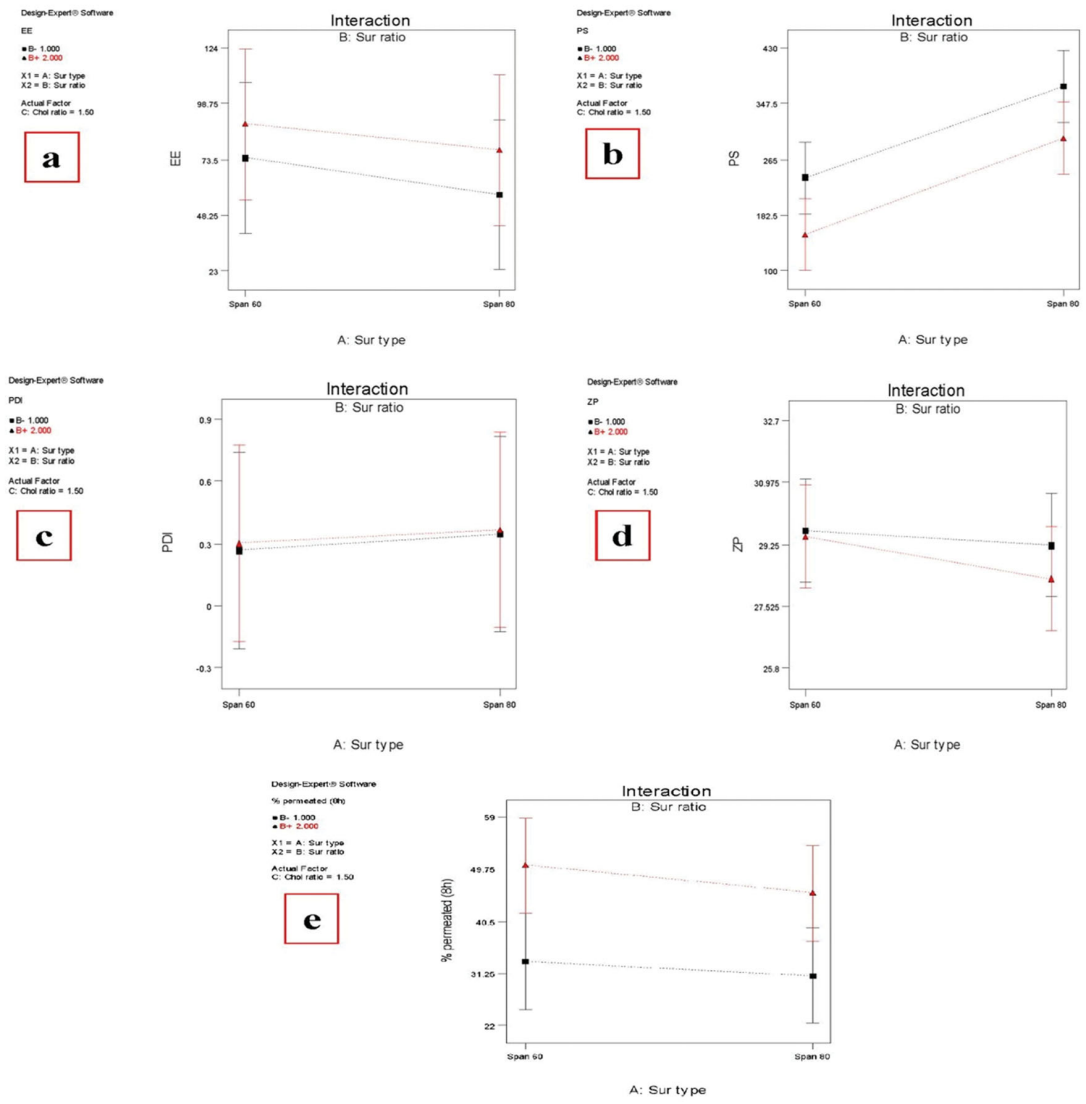
Effect of different independent factors; (X1): Ethanol concentration, (X2): PC concentration on: (a) EE%, (b) PS, (c) PDI, (d) ZP, (e) MAP % permeated (8 h) of the prepared MAP Niosomes.

The effect of different factors on the responses concerning the physicochemical properties of MAP-loaded niosomes is shown in Figure 2 and values of different responses are recorded in Table 2. The ability of niosomes to entrap a significant proportion of the drug is considered a critical parameter for optimum transdermal delivery. All the studied factors were found to have a significant effect (*P* value < .05) on the EE% of the prepared MAP-loaded niosomes.

Formulations containing Span 60 had significantly higher EE% than those containing Span 80 at the same surfactant and Chol ratio. This can be explained by the higher phase transition temperature (Tc) of Span 60 over Span 80. So, Span 60 can form vesicles with less permeable and less leaky rigid bilayers while Span 80 forms more permeable fluid bilayers. In addition, Span 80 possesses an unsaturated alkyl chain, a double bond in its alkyl chain, which makes the vesicle membrane more permeable and hampers the development of a tight vesicular membrane resulting in less EE% (Marwa et al., 2013; Vyshnavi et al., 2015).

The higher molar ratio of the surfactant resulted in a significant increase in the EE% which is in disagreement with what was reported that high surfactant concentration would increase the drug solubilization and diffusion into the aqueous media during vesicular preparation resulting in an EE% decrease (Al-Mahallawi et al., 2014), but in agreement with Imam et al., who reported that higher surfactant ratio resulted in higher entrapment efficiency (Imam et al., 2015). In our study, increasing the surfactant ratio resulted in higher EE% instead of the formation of leaky points within the niosomal vesicles and consequently decreasing the drug entrapment.

It is known that Chol has the ability to abolish the gel to the lipid phase transition of niosomal systems which could effectively prevent leakage of drugs from niosomes resulting in high EE% (Abdelbary & El-Gendy, 2008). So, it was reasonable that increasing the Chol ratio from 1 to 2 at the same surfactant ratio and type resulted in a significant EE% increase as increasing the total amount of lipids means sufficient bilayer materials for strong membrane formation and efficient drug encapsulation (Sankhyan & Pawar, 2013).

All the investigated factors were found to have a significant effect on the PS of the prepared MAP-loaded niosomes (*p* value < .05). Changing the surfactant type from Span 60 to Span 80 resulted in increase in the particle size at the same surfactant and Chol ratio. As mentioned above, Span 80 has less ability than Span 60 to form tight less permeable vesicles resulting in larger PS. A Higher Chol ratio resulted in a significant increase in the PS. This may be related to that Chol is a rigid molecule with an inverted cone shape which makes it able to be intercalated between the fluid hydrocarbon chains of the surfactant when hydrated at a temperature above the gel/liquid transition temperature resulting in an increase in the size of the vesicle (Abdelkader et al., 2014).

Also, it was reported that high Chol content will increase the hydrophobicity and the number of the bilayers membrane resulting in larger vesicular size (Al-Mahallawi, et al., 2015). Unlike Chol, a higher surfactant ratio resulted in a significantly smaller size regardless of the type of surfactant which may be related to the formation of mixed micelles (instead of niosomal vesicles), at high surfactant levels, which have a smaller particle size (Salama et al., 2012; Basha et al., 2013).

The prepared niosomes have PDI ranging from 0.253 ± 0.124 to 0.415 ± 0.131 with relatively smaller values for those formulations prepared by Span 60. A non-significant effect was found for all investigated factors on the PDI values of the prepared niosomes.

Negative zeta potential values were found for the prepared MAP-loaded niosomes, ranging from −25.82 ± 4.52 to −32.63 ± 3.84 nm with only a Chol ratio having a significant positive effect (*p* value = .0002). It was previously reported that vesicular systems with ZP value around ±30 mV are considered stable (Muller et al., 2001) which means that the prepared MAP-loaded niosomes have sufficient zeta potential values to enhance their stability.

All the studied independent variables were found to have a significant effect on the MAP cumulative percent permeated from the prepared MAP-loaded niosomes after 8 h. Among these factors, only (X2) has a positive significant effect (*p* value = .0001) as increasing the surfactant ratio resulted in higher % permeated. It is known that the presence of the nonionic surfactant will modify the structural composition of stratum corneum through partitioning into the epithelial cell membranes and disrupting the packing of membrane lipids, forming structural defects that reduce membrane integrity (Kim et al., 2020) in addition to increasing the thermodynamic activity of the drug as well as skin (Pawar et al., 2016). On the other hand, either changing the surfactant type from Span 60 to Span 80 (X1) or increasing the Chol ratio (X3) has a negative significant effect on MAP amount permeated through the skin (*P* value = .0003 and .0033 for X1 and X3 respectively). A Higher Chol ratio resulted in lower permeation. This may be related to the Chol space-filling action in which Chol molecules accommodate in the molecular cavities formed by surfactant monomers assembled into vesicles resulting in decreased permeability of the drug into the bilayers (Pawar et al., 2016). Smaller PS of niosomes formulated using Span 60 compared with those formulated using Span 80 led to an increase in the surface area available for permeation. It is worthy here to mention that the predicted *R*^2^ values were in a reasonable agreement with the adjusted *R*^2^ in all responses with the final equations in terms of coded factors for all responses were:

(8) EE%=+74.76−7.08 * A+8.88* B+5.07 * C+1.25*A*B+1.53 *A*C+1.01*B*C

(9) PS=+265.71+69.56*A−40.26*B+16.24*C+2.01*A*B−1.39*A*C+0.79*B*C

(10) PDI=+0.32+0.037*A+0.014*B−0.012*C−3.750E−003*A*B−0.014*A*C+0.014*B*C

(11) ZP=+29.16−0.40*A−0.27*B+2.59*C−0.20*A*B+0.23*A*C−0.51*B*C

(12) %permeated (8h)=+40.05−1.85 *A+7.90*B−3.70*C−0.60*A*B−0.057*A*C−0.85*B*C

### 3.4. Optimization of the prepared MAP-loaded niosomes

After all, responses were analyzed, optimized formulation was obtained by applying constraints (goals) on dependent (responses) and independent variables (factors) using Design-Expert^®^ software (version 7; Stat-Ease, Inc., Minneapolis, MN). Numerical optimization was used. Optimized formulation was selected based on the criteria of maximum EE%, minimum particle size, PDI (in range), ZP (in range), and maximum % permeated (8 h). Three solutions were obtained with the first one having a desirability of 0.938 which matched with FN3 niosomal formulation composition. So, FN3 was selected as an optimized formulation for further evaluation.

### 3.5. Transmission electron microscopy (TEM)

**Figure 3.**
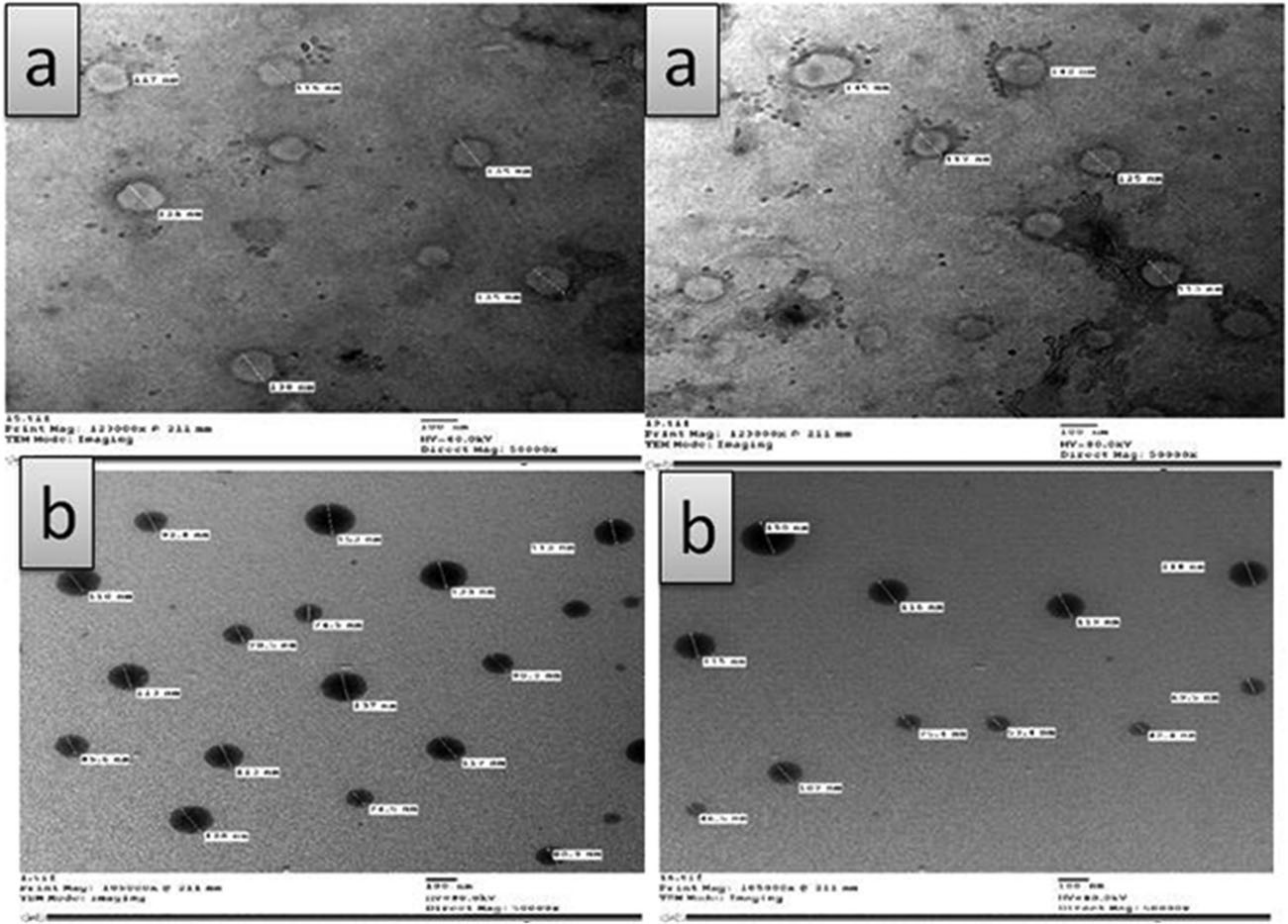
Transmission electron microscopy (TEM) photographs of: (a) MAP- loaded ethosomes (FE7), (b) MAP- loaded niosomes (FN3).

Transmission electron microscopy photographs of MAP-loaded ethosomes (FN7) showed its surface morphology as well as the existence of unilamellar vesicular structure, Figure 3(a). The MAP-loaded ethosomes appeared as discrete, round unilamellar vesicles with dark bilayer structure and light core with their size smaller than that observed by laser diffraction instrument as the later gives the hydrodynamic diameter of hydrated nanoparticles in solution, which is usually larger than the size of dried nanoparticles observed by TEM (Faisal et al., 2018). Transmission electron microscopy examination revealed that MAP-niosomes vesicles are well identified with sphere-like shapes. They have a smooth vesicle surface with uniform size, Figure 3(b).

### 3.6. Magnesium ascorbyl phosphate ethosomal and niosomal gel permeation and skin retention

**Figure 4.**
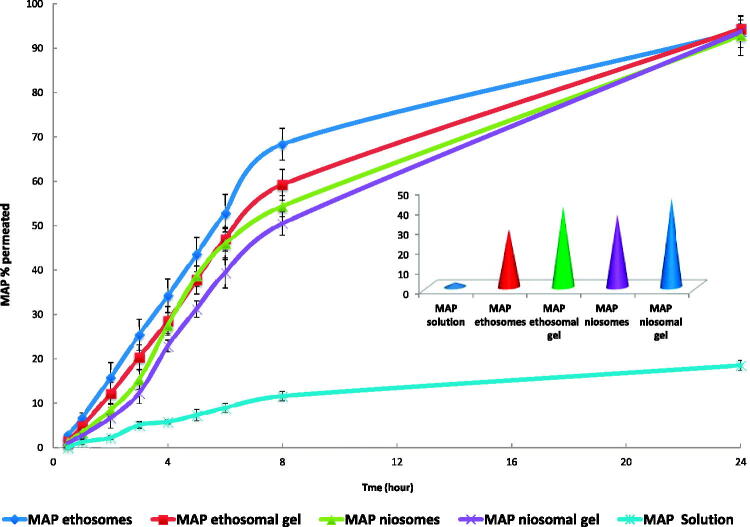
MAP skin permeation and skin retention from MAP solution, MAP ethosomes (FE7), MAP ethosomal (FE7) gel, MAP niosomes (FN3) and MAP niosomal (FN3) gel.

The percent cumulative amount of MAP permeated across excised rat skin from MAP solution, MAP ethosomes (FE7), MAP ethosomal (FE7) gel, MAP niosomes (FN3) and MAP niosomal (FN3) gel plotted against the function of time in addition to MAP cumulative amount retained in the skin after 8 hours are shown in Figure 4. All formulations have significantly higher amounts permeated compared to MAP solution. Up to 8 hours, a higher amount of MAP has permeated through rat skin from MAP ethosomes (FE7) and niosomes (FN3) compared to their gels which may be related to relative restriction of the movement of the vesicles by their gel matrix (Madhavi et al., 2019). A non-significant difference was found between all formulations concerning the MAP percent permeated after 24 h (*P* value < .05).

On the other side, MAP percent retained in the skin after 8 h were in the following order MAP niosomal (FN3) gel > MAP ethosomal (FE7) gel > MAP niosomes > MAP ethosomes with significant difference between each other (*p*-value < .05). These results indicate that carbopol gel formulations can increase water retention in the skin and thus increase its hydration and promote permeation and accumulation of the drug-loaded vesicles into the skin layers (Madhavi et al., 2019). Also, due to their hydration effect on the skin, they permit greater dissolution of actives and facilitate their transepidermal migration (Rehman & Zulfakar, 2014). For all investigated formulations, the MAP amount measured in the removed skin after 24 hours was a neglected amount which means that the entire released drug has been moved to the receptor compartment.

### 3.7. Effect of storage on selected ethosomes and niosomes formulations

Table 3 shows the results of storage of MAP ethosomal formulation (FE7) and niosomal formulation (FN3) at two different storage conditions. Results showed that storage of the ethosomal formulation at 4 °C resulted in a non-significant change (*P* value > .05) either in their EE% or PS up to 3 months with the vesicles showing no aggregation, precipitation, or color change. On the other side, storage of this formulation at room temperature showed non-significant change at the first and second month while it showed significant change at the third month (*P* value = .055 and .0499 for EE% and PS respectively) with the appearance of small visual aggregations but no sedimentation or color change. For FN3 niosomal formulation, storage at 4 °C for three months or at room temperature up to 1 month didn’t significantly affect its EE% or PS. But storage at room temperature up to 2 and 3 months resulted in a significant decrease in the EE% and increase in the PS. These results indicated that refrigeration temperature is the most optimum storage condition for both MAP ethosomes and niosomes.

**Table 3.** Effect of storage on selected ethosomes (FE7) and niosomes (FN3) formulations.

Formulation	Month	Stored at 4 °C		Stored at room temperature	
		EE%	PS (nm)	EE%	PS (nm)
FE7	0	83.43 ± 2.23	160.57 ± 13.7	83.43 ± 2.23	160.57 ± 13.7
	1	83.12 ± 1.89	163.54 ± 8.67	81.67 ± 2.34	171 ± 11.57
	2	82.63 ± 2.06	167.85 ± 10.69	79.53 ± 1.85	183 ± 9.38
	3	82.17 ± 2.18	170.52 ± 12.85	76.52 ± 1.93	189.32 ± 13.08
FN3	0	86.82 ± 4.52	138.43 ± 17.84	86.82 ± 4.52	138.43 ± 17.84
	1	86.53 ± 3.46	136.62 ± 22.54	85.48 ± 5.39	140.32 ± 19.40
	2	85.72 ± 4.08	141.31 ± 19.42	82.57 ± 3.62	153.72 ± 23.43
	3	85.03 ± 3.94	141.54 ± 24.62	81.44 ± 4.51	159.89 ± 22.24

EE: Entrapment Efficiency, PS: Particle size.

### 3.8. Efficacy of magnesim ascorbyl phosphate ethosomal and niosomal gel carriers in treating melasma using ANTERA 3 D^®^ camera

#### 3.8.1. Demographic data and clinical characteristics of studied subjects

The present study included 40 individuals seeking treatment of melasma and facial hyperpigmentation from those who attended to the Dermatology outpatient clinic of Ain Shams University Hospitals from February 2020 to January 2021. Twenty-three women (57.5%) and 17 men (42.5%) were recruited. The age of patients ranged from 25 to 55 years old, with a mean age of 41.90 years old ± 9.097 SD and of skin types II (1 patient= 2.5%), III (24 patients= 60%) and IV (15 patients= 37.5%). All patients gave a history of regular use of sunscreens. 19 patients (47.5%) were housewives, and 21 (52.5%) patients were office-based working with minimal sun exposure, Table 4.

**Table 4.** Demographic and clinical data.

	*N* = 40
Gender	
Women	23 (57.5%)
Men	17 (42.5)
Age	
Mean ± SD	41.90 ± 9.097
Range	25–55
Skin type	
II	1 (2.5%)
III	24 (60%)
IV	15 (37.5)
Rt side Average melanin level before Treatment	
Mean ± SD	0.799 ± 0.1190
Range	0.5–1
Rt side Average melanin level After 1 month	
Mean ± SD	0.776 ± 0.1158
Range	0.5–1
Rt side Average melanin level After 6 months	
Mean ± SD	0.748 ± 0.1608
Range	0.1–1
Lt side Average melanin level before treatment	
Mean ± SD	0.792 ± 0.1321
Range	0.6–1
Lt side Average melanin level After 1 month	
Mean ± SD	0.772 ± 0.1234
Range	0.6 – 1
Lt side Average melanin level After 6 months	
Mean ± SD	0.758 ± 0.1619
Range	1

#### 3.8.2. Evaluation of treatment response on the right side (niosomal gel treated side)

The average melanin level at the baseline ranged from 0.5 to 1 with a mean average of 0.799 ± 0.1190 which decreased after one month of treatment to a mean of 0.776 ± 0.1158, with an average change of −0.023 ± 0.0758 and a percentage of change of about −2.379 ± 11.1785. This decrease was not statistically significant with *P* value = .062. At the 6 months follow-up, it decreased to a range of 0.1–1 and an average of 0.748 ± 0.1608, the change from the baseline was −0.051 ± 17.3829 with a percentage of −5.670 ± 17.3829 and this change was statistically significant with *P* value = .033.

#### 3.8.3. Evaluation of treatment response on the left side (ethosome gel treated side)

The average melanin concentration at the baseline ranged from 0.6 to 1 with an average of 0.792 ± 0.1321, this decreased after one month of treatment to 0.772 ± 0.1234, with an average change of −0.020 ± 0.0474 and a percentage of change of −2.24 ± 6.627. This decrease was statistically significant with *P* value = .01. At the 6 months follow-up, it decreased to a range of 0.1–1 and an average of 0.758 ± 0.1619, the change from the baseline was −0.034 ± 0.1302 with a percentage of −3.730 ± 15.3345 and this change wasn’t statistically significant with *P* value = .105.

#### 3.8.4. Comparative study

**Figure 5.**
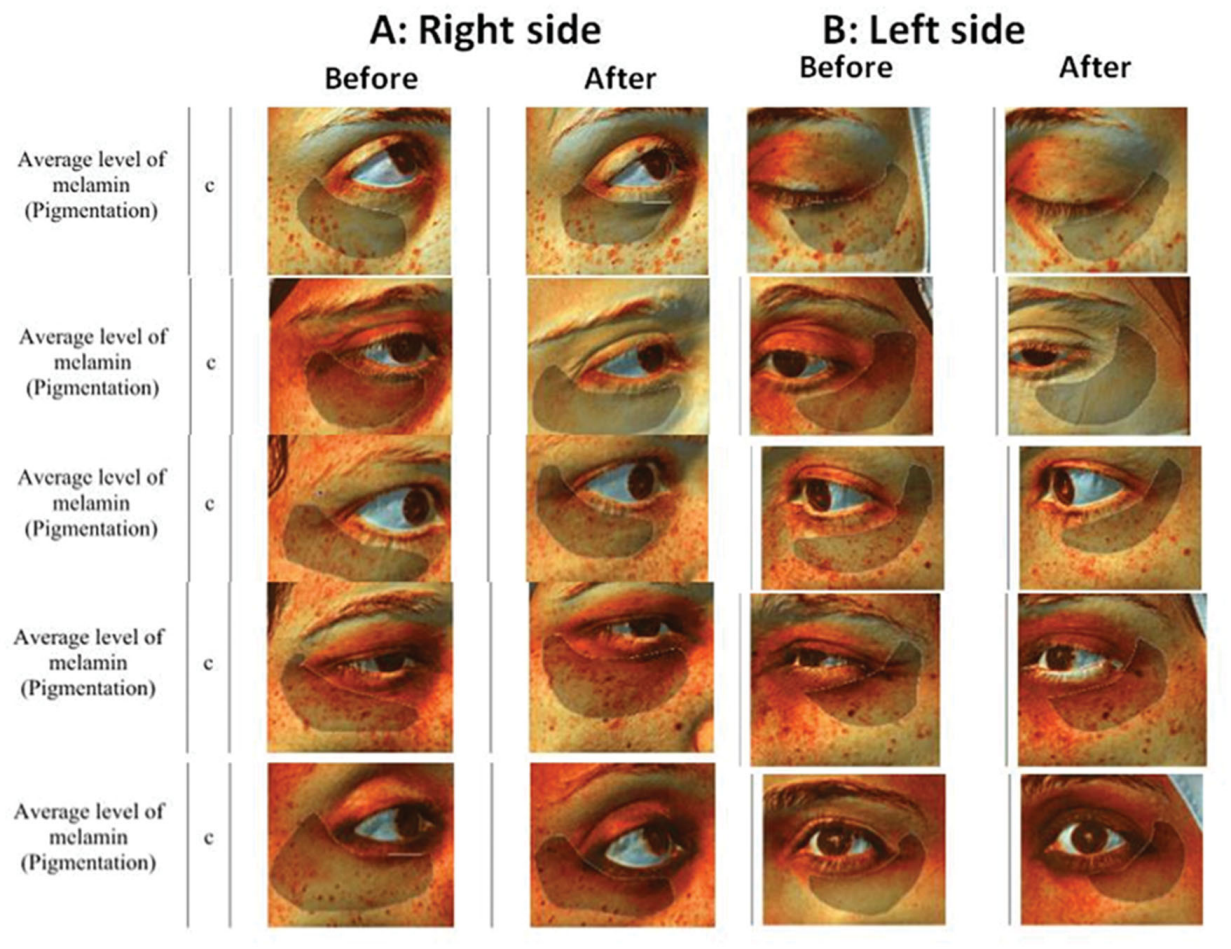
Samples of Antera 3D^®^ Camera photos of before and after treatment with: (A) Right side (MAP niosoma gel) and (B) Left side (MAP ethosoal gel).

Comparing the right (niosome) side to the left (ethosome) side resulted in a non-significant difference between the average melanin concentrations on the two sides at the baseline, *P* value = .723. Although the average change on the right side was higher than the average change on the left side, this difference was not statistically significant either after 1 month nor 6 months, *P* value= .945 and .623 respectively. Samples of Antera 3 D^®^ camera photos before and after treatment are represented in Figure 5.

Concerning the reported adverse events, 8 patients reported erythema on the niosome side compared to 11 patients that reported mild erythema on the ethosome side, none of our patients complained of itching or burning, erythema was mild and none of the patients stopped treatment because of it.

In the current study, the average melanin level mode measured by Antera 3 D^®^ camera was used for assessment of skin pigmentation. Antera 3 D^®^ camera uses an innovative optical method and complex mathematical algorithms to obtain three-dimensional images. This makes it possible to extract numerical objective data from images, which will allow quantifying the efficacy of treatments and monitoring changes over time. The Antera 3 D^®^ camera also analyzes and measures topographic features of the skin (such as texture, wrinkles and fine lines) (Kang et al., 2002; Stamford, 2012).

Several techniques have been tried for enhancing ascorbic acid penetration and thus improving its whitening results. Back in 2001, Smith tried lactic acid 8.8% in combination with ascorbic acid at 1% concentration. Their study showed 4% improvement in whitening after 3 months of treatment which was statistically better than ascorbic acid alone caused no difference in the same study. Although this proves that lactic acid can enhance ascorbic acid skin penetration, yet the results were not convenient (Smith, 1999). These results were close to both gels used in the current study and both studies used objective assessment of melanin level- where they used skin color-Minolta Meter. Smith suggested that, although a 4% change might sound slight, yet it’s not only statistically significant but clinically significant as well, as the baseline concentration is the artificially high baseline. Other chemical peels have been used to enhance cutaneous penetration of ascorbic acid as trichloroacetic acid (Murtaza et al., 2016).

Recently, microneedling has also been tried with ascorbic acid to overcome its poor skin penetration ability. Menon et al found 3.7% and 12.3% improvements using the melasma area severity index (MASI) (Menon et al., 2020). Although the improvement percentage is higher than our current study, yet objective assessments are usually lower than subjective assessments. Subjective assessment of MAP- aspasomal cream using hemi Melasma Area and Severity Index score (hemi-MASI) was published earlier as a part of our research and showed an average improvement of 27.8% at 6 weeks and 47.36% at twelve weeks (Aboul-Einien et al., 2020). This indicates that an objective assessment showing 5% and 3% improvement would be significantly clinically visible.

In the current split-face comparative study, both niosomal and ethosomal gels of MAP showed efficacy in treating melasma as measured objectively using melanin level assessment by the ANTERA 3 D^®^ camera. However, on comparing both sides, and although the final outcome showed a non-statistically significant difference between the two sides, it was interesting to find that the significant change took one month in the case of ethosomes then non-significant change at the 6 months follow up, while it took 6 months for niosomes to obtain a significant result with greater change in melanin concentration (5% compared to 3%). It will be interesting to study combining the application of both formulations as it can have a synergistic effect as one of them can induce rapid results while the other can improve the persistence of the results.

## 4. Conclusion

Topical nanosized ethosomal and niosomal delivery systems containing MAP were formulated and optimized using factorial design. For the developed ethosomal and niosomal formulations, all the studied independent factors were found to have a significant effect on the EE%, particle size and % drug permeation of the prepared formulations. The optimized ehosomal formulation (EF7) and niosomal formulation (NF3) were selected on the basis of maximum EE% and % dug permeated with low particle size. Both the ethosomal and niosomal gels had significantly lower ex vivo % MAP permeated than their ehosomal and niosomal formulations, but higher amounts were retained into the skin after 8 hours allowing controlled and effective drug delivery across the skin layers. When the MAP ethosomal and niosomal gels were evaluated clinically for melasma treatment, both showed significant improvement of melasma, with faster response of the ethosomes and more long-lasting effect of the niosomes with minimal side effects. We suggest that combining the two formulations might yield faster and more lasting results.

## Data Availability

The authors confirm that the data supporting the findings of this study are available within the article.
